# Multiple-Dynamic-Bond Cross-Linked Injectable Antibacterial Hydrogel Sealants with Self-Healing for Wound Healing

**DOI:** 10.3390/gels12040340

**Published:** 2026-04-19

**Authors:** Tingting Wei, Yunrui Cao, Shuo Yang, Yu Song, Yanjun Liu, Hu Hou, Jie Xu, Changhu Xue

**Affiliations:** 1 State Key Laboratory of Marine Food Processing & Safety Control, College of Food Science and Engineering, Ocean University of China, No. 1299, Sansha Road, Qingdao 266404, China; ttwei621@163.com (T.W.); yangshuo19988183@163.com (S.Y.); songyu@ouc.edu.cn (Y.S.); liuyanjun@ouc.edu.cn (Y.L.); houhu@ouc.edu.cn (H.H.); 2 Optoelectronic Information Center, Taizhou Institute of Zhejiang University, Taizhou 318000, China; caoyunrui2024@zju.edu.cn; 3 Sanya Ocean Institute, Ocean University of China, Sanya 572000, China; 4 Qingdao Marine Science and Technology Center, Qingdao 266235, China

**Keywords:** aldehyde carboxymethylated agarose, biomineralization, injectable hydrogel, multiple-dynamic-bond crosslinked network self-healing, wound healing

## Abstract

Chronic wounds resulting from bacterial infection remain one of the main challenges in clinical practice. There is a pressing need to develop an injectable hydrogel sealant with multifunctional properties, including remodeling capabilities, self-healing, painless removal, and antibacterial activity, to promote tissue remodeling. In this work, aldehyde carboxymethylated agarose (ACMA) is employed for the first time as a bio-template. Dopamine (DA) is introduced onto the ACMA template via a reversible Schiff-base reaction, endowing it with biomineralization properties to synthesize DA-modified ACMA-Ag nanoparticles (ACMA-DA-Ag). Further, the prepared ACMA-DA-Ag, which possesses both antibacterial activity and injectable behavior, is incorporated into a guar gum hydrogel through the formation of borate/diol bonds, thereby forming a multiple-dynamic-bond crosslinked network. This hydrogel demonstrates adequate mechanical strength, injectability, remodeling capabilities, and self-healing performance. It can reassemble into a new hydrogel within 4 ± 0.6 min upon simple physical contact, and supports tissue adhesion. Furthermore, the hydrogel effectively covers irregular-shaped wound and can be removed without causing secondary injury. More importantly, this multifunctional hydrogel is cost-effective, easy to synthesize, and simple to use, significantly accelerating skin regeneration and promoting the formation of skin appendages, such as hair follicles. The outcome of this research not only serves a tissue sealant for wound healing, but also presents a new strategy for creating novel polysaccharide-based biomaterials.

## 1. Introduction

As the body’s primary defense barrier, the skin is highly vulnerable to external environmental stimuli such as cuts, impacts, and friction, resulting in skin trauma [1,2,3,4]. While minor skin lesions typically heal on their own, more severe injuries—particularly chronic wounds—are often complicated by infections due to microbial and inflammation invasion, which can further cause more serious consequences [5,6,7]. Rapid wound closure is essential for preventing microbial invasion, promoting tissue regeneration, and accelerating wound healing. As a result, manifold medical adhesive bandages, sutures, and tension strips have been widely used in clinical practice [8,9]. Nevertheless, postoperative complications, including wound infection and foreign body reaction, continue to arise, primarily due to the absence of a favorable moist microenvironment at the wound site to support cell proliferation [10,11]. In addition, secondary injuries cannot only prolong the healing time but also increase the risk of infection, causing additional pain and discomfort for the patient [12]. Despite significant advancements in the development of various wound dressings—such as membranes, nanofibers, modified gauzes, and hydrogels—with unique biochemical functions, creating materials that can simultaneously accelerate wound closure and promote rapid healing remains a major challenge [13,14,15]. Hydrogels, due to their similarity to biological soft tissues and their ability to maintain a moist microenvironment, have been proposed as an effective alternative for restoring damaged skin tissue [16,17,18,19,20]. Therefore, developing an efficient hydrogel-based material focusing on accelerating wound closure and healing in a sutureless manner is required. Recently, injectable hydrogels have garnered significant attention in wound management due to their ability to fully fill irregular, deep wounds, and resist biofouling and bacterial adhesion [8,10].

Agarose, a natural neutral polysaccharide derived from red algae, can form a stable gel by cross-linking. Due to its ideal gelling properties, ability to maintain a moist microenvironment, excellent biocompatibility, and unique mechanical characteristics, it has been extensively developed and utilized as a functional wound dressing [21,22]. Nevertheless, its relatively low chemical complexity limits its application in many biochemical reactions. Introducing functional groups into agarose is an effective method to modify its physicochemical attributes [22]. Modifications such as the introduction of carboxymethyl and aldehyde groups can effectively improve the properties of agarose. Furthermore, both of these groups are reactive, allowing them to interact with various chemicals and thereby introduce novel functionalities to the material [22].

To produce multifunctional hydrogel wound dressings, introducing metal-based nano-materials into nano-composite hydrogels is a promising strategy [23,24,25,26]. Biomineralization can effectively combine organic macromolecules with inorganic substances, leading to the formation of uniform and stable metal-based nanoparticles. Recent studies have demonstrated the production of various nanomaterials, such as Ag_2_S, Au and Ag nanoparticles, by using organic macromolecules as soft templates via biomineralization strategies [27,28,29,30]. Dopamine (DA) has been grafted onto macromolecules like sodium alginate, hyaluronic acid, and chitosan to impart multiple properties to these materials [31,32,33]. However, the utilization of the strong reducibility of DA catechol groups has rarely been reported. In this work, we incorporated these groups into the biomaterial backbone via dynamic imine bonds to construct reversible gels while simultaneously imparting biomineralization capabilities to the system. This design effectively enhances the multifunctional utilization of DA in wound dressings.

**Scheme 1:**
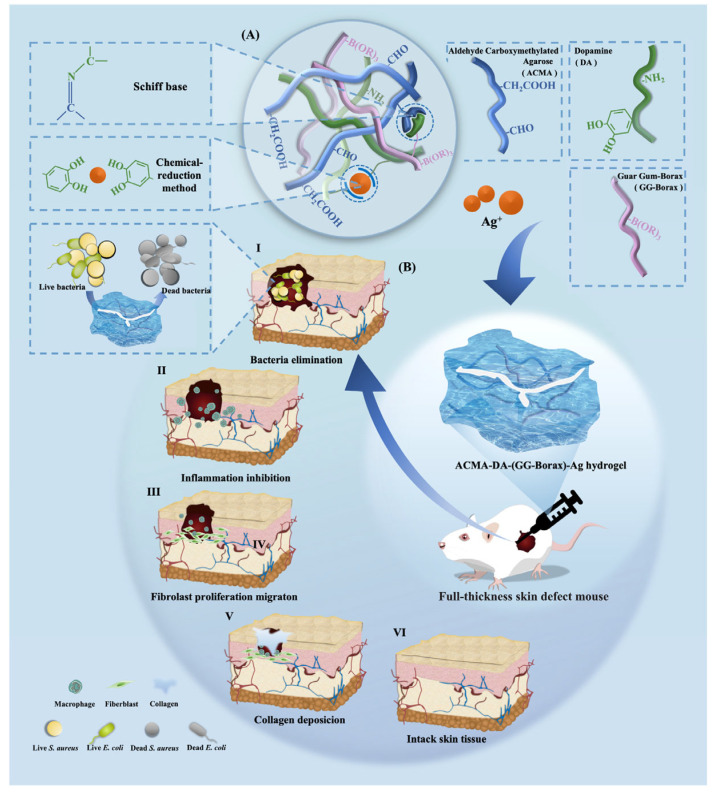
(**A**) Schematic and mechanism of preparing the ACMA-DA-(GG-Borax)-Ag injectable antibacterial hydrogel and (**B**) application of the ACMA-DA-(GG-Borax)-Ag injectable antibacterial hydrogel to wound healing.

Herein, we have developed a multifunctional multi-network hydrogel, referred to as ACMA-DA-(GG-Borax)-Ag tissue sealant, designed for the treatment of irregular wounds without the need for sutures. As shown in Scheme 1, we first oxidized carboxymethylated agarose (CMA) to serve as the basic backbone, followed by grafting DA via the dynamic Schiff-base crosslinking.

The aldehyde carboxymethylated agarose (ACMA)-DA was utilized as a biomineralization agent to prepare an aqueous precursor of ACMA-DA-Ag, eliminating the need for additional reducing agents and stabilizers. This streamlined approach simplifies the preparation process. Next, guar gum (GG) and borax were added to the aqueous precursor of ACMA-DA-Ag, forming the ACMA-DA-(GG-Borax)-Ag tissue sealant. The injectable adhesive hydrogel can be easily administered to fully fill irregular wounds, adapt to tissue movement, and exhibit outstanding remodeling, self-healing and antibacterial properties. Meanwhile, the hemolysis and cytotoxicity experiments revealed good biocompatibility of the tissue sealant. Furthermore, in vivo wound closure assessments demonstrated that the tissue sealant effectively prevents infection, attributed to the inherent antibacterial properties of the Ag NPs. All the results implied that the multifunctional ACMA-DA-(GG-Borax)-Ag tissue sealant demonstrated advantages and excellent application potential for selecting optimal wound dressings.

## 2. Results and Discussion

### 2.1. Fabrication and Characterization of the ACMA-DA-(GG-Borax)-Ag Hydrogel

**Figure 1:**
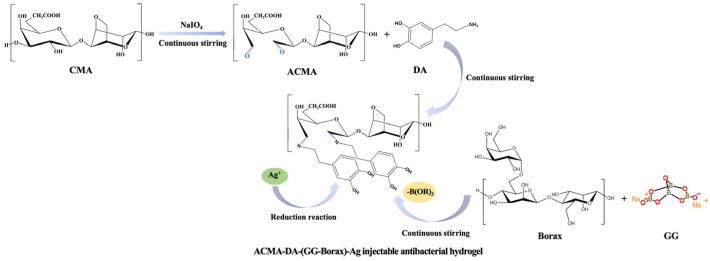
The synthesis route of the ACMA-DA-(GG-Borax)-Ag injectable antibacterial hydrogel.

The formation procedure of the ACMA-DA-(GG-Borax)-Ag hydrogel is depicted in Scheme 1 and Figure 1. Initially, ACMA was synthesized using NaIO_4_ as the oxidant, which served as the basic bio-template. Prior to hydrogel formation, under room-temperature conditions with continuous stirring, the aldehyde groups (-CHO) on ACMA were linked with -NH_2_ on DA via a reversible Schiff-base reaction to form -RC=N- bonds. Subsequently, Ag^+^ antibacterial agents were added, and the biomineralization property of DA enabled the efficient reduction of Ag^+^ to Ag NPs, which were uniformly loaded in the gel. Finally, the gel was further reinforced by the addition of GG and borax, which formed dynamic covalent -B(OR)_3_ linkages (i.e., the cations of GG combined with Borax to form a cross-linked structure), assisting the formation of the ACMA-DA-(GG-Borax)-Ag injectable antibacterial hydrogel. This was cross-linked through multiple dynamic bonds, including -RC=N-, -B(OR)_3_, and hydrogen bonds.

The characterization and analysis of the CMA were shown in Figure S1 in the Supporting Information . Fourier transform infrared spectroscopy (FTIR) and ^1^H-Nuclear magnetic resonance (^1^H-NMR) were performed to investigate the functional groups of ACMA. As presented in Figure S2A, the FTIR spectrum of ACMA, compared to that of CMA, showed a red shift and an increase in peak intensity for the C=O stretching vibration. This could be attributed to the formation of aldehyde groups. The adjacent diols in the D-galactose structural units of CMA were oxidized and cleaved, thereby forming two aldehyde groups. As shown in Figure S2B, a new chemical shift appeared at δ 9.5–10.0 ppm in the ^1^H-NMR spectrum of ACMA, which could be assigned to the aldehyde groups. However, the relatively weak intensity of this shift implied a low concentration of aldehyde groups, with the oxidation rate calculated to be 7% based on the integral area.

Upon mixing and stirring the ACMA solution with the DA solution, a low-viscosity yellowish solution was obtained (Figure S3). The yellow coloration is attributed to the C=N double bond, a hallmark of Schiff bases, thereby indirectly confirming the formation of Schiff-base linkages [34].

**Figure 2:**
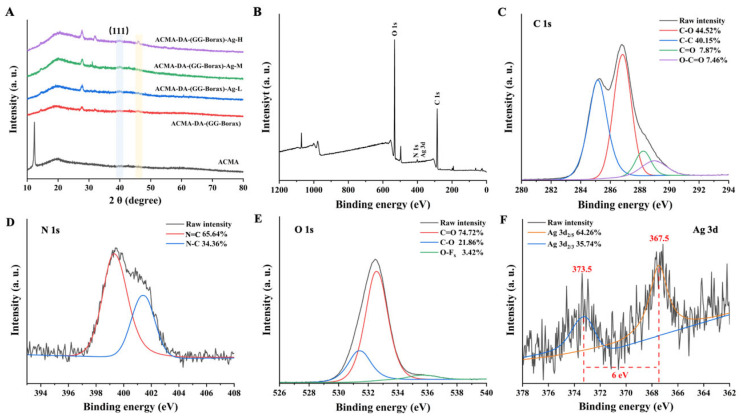
Spectra of the ACMA-DA-(GG-Borax)-Ag hydrogel. (**A**) XRD patterns; (**B**) XPS spectrum; (**C**–**F**) XPS high-resolution spectra: C 1s, N 1s, O 1s and Ag 3d peaks.

The X-ray diffraction (XRD) patterns of ACMA, ACMA-DA-(GG-Borax) and ACMA-DA-(GG-Borax)-Ag hydrogel are shown in Figure 2A. Compared to ACMA and ACMA-DA-(GG-Borax), ACMA-DA-(GG-Borax)-Ag hydrogel displayed an additional peak at 39.36°, which was reflected as the (111) crystal face of Ag NPs, further identifying that Ag NPs were successfully embedded into the hydrogel. Meanwhile, the characteristic peak in the Ag (200) crystal face appeared at 45.98°. However, the characteristic peak in the Ag (220) crystal face at around 63.3° could not be observed, possibly due to shielding by the ACMA.

The elemental composition of C 1s, N 1s, O 1s and Ag 3d was analyzed using X-ray photoelectron spectroscopy (XPS) survey spectra (Figure 2B). As shown in Figure 2C, the C 1s peaks were resolved into four distinct peaks at 285.1, 286.8, 288.2 and 288.9 eV, corresponding to C-C, C-O, C=O and O-C=O, respectively. The N 1s peak in the high-resolution XPS spectrum appeared at 399.2 and 401.3 eV, belonging to C=N and N-C (Figure 2D). Furthermore, the O 1s peaks were deconvoluted into three peaks at 531.3, 532.5 and 535.6 eV, corresponding to C=O, C-O and O-Fx, respectively (Figure 2E). These results indicated that the -NH_2_ groups on DA were successfully cross-linked with the -CHO groups on ACMA via -RC=N- bonds. Furthermore, in Figure 2F, doublet peaks at 373.5 and 367.5 eV appeared in the high-resolution XPS spectrum of Ag 3d, corresponding to Ag 3d_5/2_ and Ag 3d_3/2_, respectively. These results confirmed that the embedded Ag element existed in the elemental state (Ag^0^), agreeing well with the XRD analysis.

**Figure 3:**
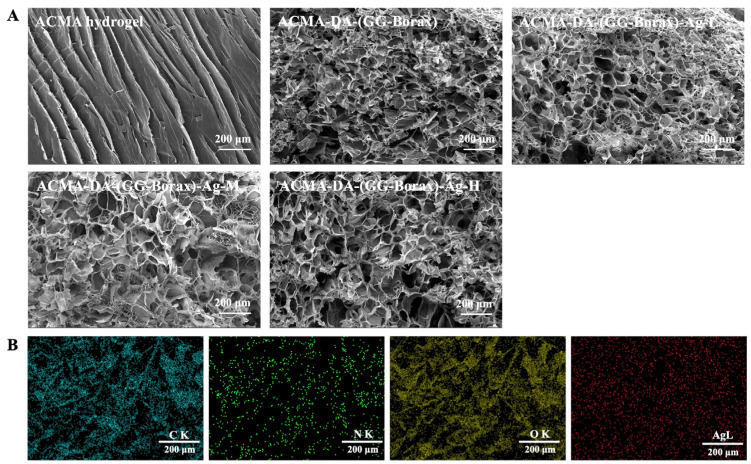
SEM image (**A**) and elemental mapping images (**B**) of C, N, O and Ag of the ACMA-DA-(GG-Borax)-Ag hydrogel.

The morphology of the injectable antibacterial hydrogel was characterized by a scanning electron microscope (SEM, Figure 3A). The results revealed that the prepared hydrogel had a dense porous structure, with variations in pore size likely attributed to the increased cross-linking density within the gel network. The SEM mapping in Figure 3B clearly disclosed the uniform distribution of C, N, O and Ag in the obtained ACMA-DA-(GG-Borax)-Ag hydrogel.

As illustrated in Figure S4A, all hydrogels exhibited a rapid initial swelling process. The ACMA-DA-(GG-Borax)-Ag hydrogel achieved a maximum swelling degree of approximately 140%, which enabled it to maintain a moist wound microenvironment and absorb excess wound exudate. Nevertheless, the potential risks and challenges associated with the swelling of injectable hydrogels in confined spaces should not be overlooked [8]. These include tissue damage induced by mechanical pressure, as well as compromised structural integrity and functional failure [8,23,29]. In spatially restricted environments, hydrogel expansion might exert sustained and excessive mechanical pressure on surrounding tissues, potentially causing local ischemia, nerve compression, or functional impairment of adjacent vital structures [8,23,29]. Furthermore, hydrogel swelling might be incomplete or non-uniform under physical confinement, thereby impairing its exudate absorption efficiency [29]. As shown in Figure S4B, all hydrogels exhibited a sustained degradation profile over a 48 h incubation period, which helped mitigate the risk of potential toxicity from rapid material degradation.

### 2.2. Self-Healing, Reshaping Property, Injectability and Tissue Adhesion

**Figure 4:**
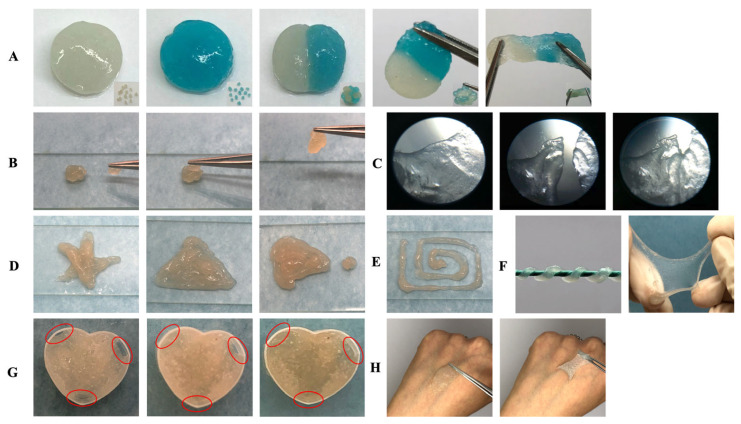
Photograph of the ACMA-DA-(GG-Borax)-Ag hydrogel. (**A**) Self-healing (stained with blue dye); (**B**) adhesion; (**C**) gel interface state under microscope; (**D**) re-moldability; (**E**) injectivity; (**F**) distortion and stretchability; (**G**) fluidity; (**H**) removal.

Since hydrogels with self-healing properties could significantly extend the service life of wound dressings [34,35,36], a macro-healing experiment was adopted to evaluate the self-healing ability of the ACMA-DA-(GG-Borax)-Ag hydrogel (Figure 4A). In this experiment, two cylindrical hydrogel samples were cut into two halves, re-adhered, and placed at room temperature. The ACMA-DA-(GG-Borax)-Ag hydrogel could rejoin into a new hydrogel with uneven color within 4 ± 0.6 min only through physical contact. At the same time, no cracks appeared when the above hydrogel was stretched with tweezers, indicating its excellent self-healing properties. In addition, a similar effect was observed when the hydrogel was fragmented into smaller particles (Supplementary Figure S4A). As shown in Movie S1 and Figure 4B, the ACMA-DA-(GG-Borax)-Ag hydrogel could rapidly adhered to a hydrogel weighing five times its own weight and be lifted. To further observe the self-healing process, an optical microscope was used. After physical contact, the two halves of the hydrogel quickly adhered together and self-healed into a complete cylinder within 5 min, with no visible cracks in the repair under the microscope (Figure 4C). As shown in Figure 4D, the ACMA-DA-(GG-Borax)-Ag hydrogel exhibited sufficient flexibility and could be reshaped into various complex 3D shapes, including a pentagram, triangle, exclamation mark, etc. These results collectively suggest that the ACMA-DA-(GG-Borax)-Ag hydrogel, with multiple dynamic bonds through physical cross-linking, could dissociate quickly under external force and rapidly rebuild once the external force was removed. Movie S2 and Figure 4E showed that the ACMA-DA-(GG-Borax)-Ag hydrogel could be continuously extruded from a 5 mL syringe without a needle, demonstrating its promising potential for biomedical applications. Figure 4F showed that the ACMA-DA-(GG-Borax)-Ag hydrogel could be twisted into various shapes and stretched into a film. As shown in Figure 4G, the hydrogel exhibited fluidity, enabling it to effectively fill gaps. Furthermore, the ACMA-DA-(GG-Borax)-Ag hydrogel not only adheres strongly to the surface of the back-of-hand skin, showcasing excellent adhesion, but can also be peeled off without leaving any residue or causing secondary damage to the skin (Movie S3 and Figure 4H). Based on the above results, it could be inferred that the reversible dynamic covalent bonds (-RC=N-, -B(OR)_3_) and physical non-covalent bonds (hydrogen bonds) in the hydrogel were the key to its sufficient self-healing property, reshaping property, injectability, tissue adhesion, etc.

### 2.3. Rheological Properties of the ACMA-DA-(GG-Borax)-Ag Hydrogel

**Figure 5:**
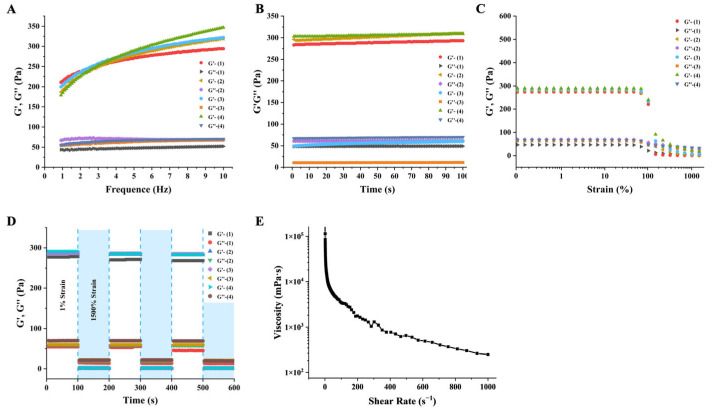
(**A**) Rheological frequency-sweep curves; (**B**) rheological time-sweep curves; (**C**) rheological strain amplitude sweep curves; (**D**) cyclic strain step sweep curves; (**E**) viscosity curve with increasing shear rat of the ACMA-DA-(GG-Borax)-Ag hydrogel (G-(1) ACMA-DA-(GG-Borax); G-(2) ACMA-DA-(GG-Borax)-L; G-(3) ACMA-DA-(GG-Borax)-M; G-(4) ACMA-DA-(GG-Borax)-H).

Subsequently, rheological tests were conducted to evaluate the stability and injectability of the ACMA-DA-(GG-Borax)-Ag hydrogel. As depicted in Figure 5A,B, within the specified frequency range (1–10 Hz) and time range (0–100 s, 5 Hz), the storage modulus (G′) of the ACMA-DA-(GG-Borax)-Ag hydrogel (at Ag NPs concentrations of 0.05, 0.1, and 0.2 μg/mL, respectively) was consistently higher than the loss modulus (G″). Moreover, no significant changes in the modulus were observed with increasing Ag NPs content. These findings indicated that the ACMA-DA-(GG-Borax)-Ag hydrogel could maintain a stable gel state. Furthermore, the increase in G’ with rising frequency suggested that physical interactions, rather than covalent bonds, primarily govern the network behavior. The strain amplitude sweep curve revealed that the ACMA-DA-(GG-Borax)-Ag hydrogel remained in a gel state (G′ > G″) within the low strain range (1–100%), but transitioned to a sol state when the strain exceeded 1000%. This phenomenon suggested that the decomposition of -RC=N- and -B(OR)_3_ bonds, along with the dissociation of hydrogen bonds, contributed to the sol–gel transition as strain increased (Figure 5C). In the step strain test, the ACMA-DA-(GG-Borax)-Ag hydrogel quickly recovered its original G′ value after switching from high strain (1500%) to low strain (1%), confirming its excellent self-healing ability. This rapid recovery was attributed to the dynamic reorganization of -RC=N- and -B(OR)_3_ covalent bonds, as well as hydrogen bonds, within the hydrogel network (Figure 5D). Additionally, the viscosity of the hydrogel decreased with increasing shear rate (Figure 5E), exhibiting typical shear-thinning behavior. The zero-shear viscosity (η_0_) was 1 × 10^5^. Taken together, these results highlighted that the dynamic -RC=N-, -B(OR)_3_ bonds and hydrogen bonds within the ACMA-DA-(GG-Borax)-Ag hydrogel were essential for maintaining its structural stability and conferring its superior self-healing ability.

### 2.4. In Vitro Antimicrobial Properties of the ACMA-DA-(GG-Borax)-Ag Hydrogel

**Figure 6:**
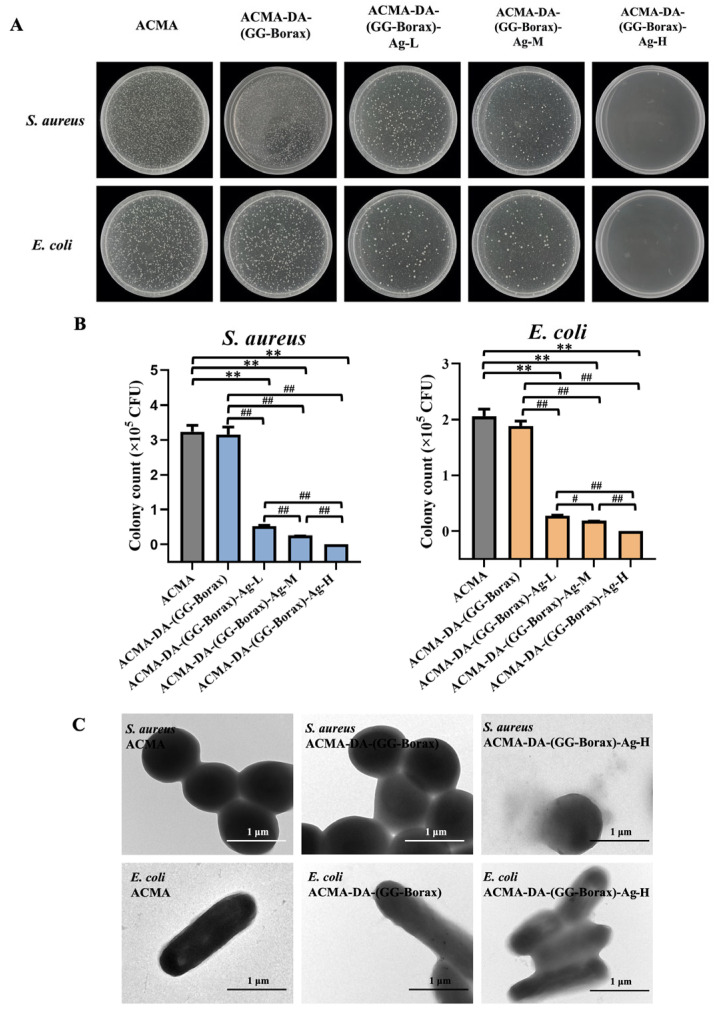
Antibacterial characterization of the ACMA-DA-(GG-Borax)-Ag hydrogel against *S. aureus* and *E. coli*. (**A**) Photographs of agar plates smeared with *S. aureus* and *E. coli* after treatment with different groups and (**B**) corresponding statistical data of colonies; (**C**) TEM images showing the morphological changes in *S. aureus* and *E. coli* after incubation with hydrogel. (*n* = 6; ** *p* < 0.01; ^#^ *p* < 0.05, ^##^ *p* < 0.01).

The excellent inherent antibacterial properties of Ag NPs inserted in the hydrogel prompted us to evaluate the hydrogel’s antibacterial activity against *Staphylococcus aureus* (*S. aureus*) and *Escherichia coli* (*E. coli*), two common pathogens responsible for most infections. As shown in Figure 6A, significant reductions in the colony numbers of both *S. aureus* and *E. coli* were observed in the ACMA-DA-(GG-Borax)-Ag hydrogel-treated groups compared to the control and ACMA-DA-(GG-Borax) groups. Moreover, as the concentration of Ag NPs increased, the total number of colonies further decreased, indicating that the hydrogel exhibited significant antibacterial activity due to the presence of Ag NPs. Further, the antimicrobial performance of the hydrogel was characterized by the colony-forming unit (CFU) method. As illustrated in Figure 6B, the calculated antibacterial rates of the ACMA-DA-(GG-Borax)-Ag-H hydrogel against both *S. aureus* and *E. coli* were all 100%, whereas the CFU numbers of the above two bacteria decreased by more than three orders of magnitude.

The cell morphology and membrane integrity of bacteria treated with different hydrogels were observed by TEM to further investigate the antibacterial process of the ACMA-DA-(GG-Borax)-Ag (Figure 6C). In the ACMA and ACMA-DA-(GG-Borax) groups, both bacterial strains still maintained an intact, regular cellular morphology with a smooth membrane. In comparison, following treatment with the ACMA-DA-(GG-Borax)-Ag hydrogel, notable morphological changes were observed, including surface wrinkling of the bacterial envelopes. Furthermore, it was clearly observed that the bacterial walls and membranes were disrupted, causing cytoplasmic leakage and possible DNA damage. These results confirmed that the morphology of the above bacteria was consistent with the antibacterial mechanism of Ag NPs [37,38], and the ACMA-DA-(GG-Borax)-Ag-H exhibited the most pronounced antibacterial effect.

### 2.5. Investigation of the Biocompatibility of the ACMA-DA-(GG-Borax)-Ag Hydrogel

**Figure 7:**
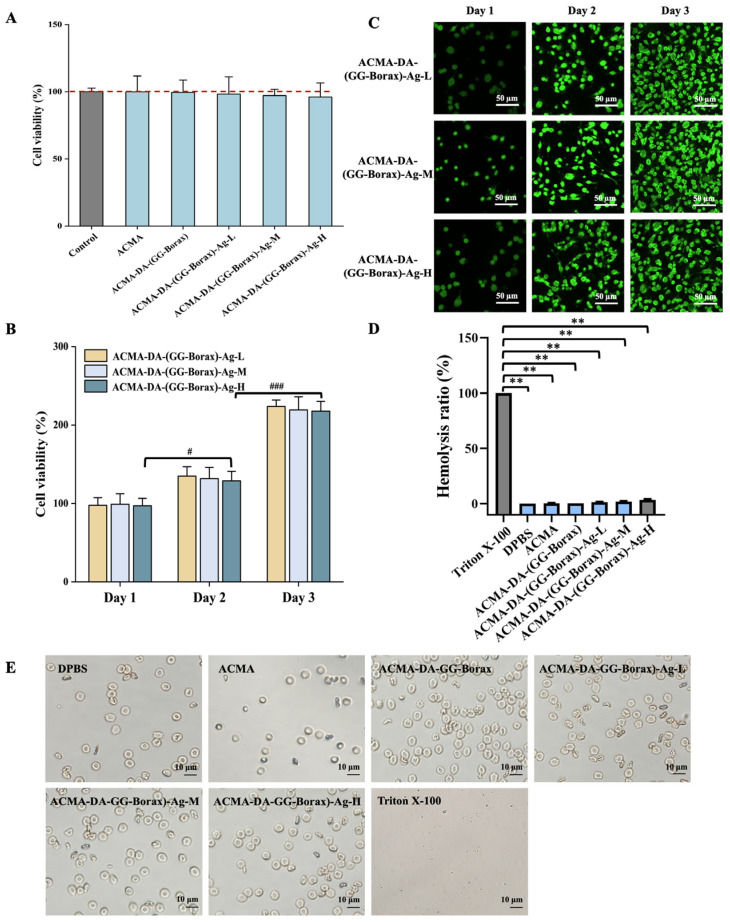
Cytocompatibility and hemocompatibility analysis of the ACMA-DA-(GG-Borax)-Ag hydrogel. (**A**) Statistical data from the MTT assay; (**B**) statistical data from the live/dead assay; (**C**) live/dead staining fluorescent images; (**D**) statistical data from the hemolysis assay; (**E**) micrographs of the hemolysis assay. (*n* = 6; ** *p* < 0.01; ^#^ *p* < 0.05, ^###^ *p* < 0.001.).

Biocompatibility, encompassing both cytocompatibility and hemocompatibility, is a prerequisite for the biomedical applications of hydrogels [39,40]. Regarding cytocompatibility, as shown in Figure 7A, all groups showed negligible toxicity to L929 fibroblasts after co-incubation with different hydrogel-conditioned media for 24 h. No significant differences were observed between the control group and the ACMA-DA-(GG-Borax) groups, both with and without Ag NPs. Even at the highest Ag NPs concentration, 96.18% of the L929 cells remained viable. To present additional proof, we co-cultured the ACMA-DA-(GG-Borax)-Ag-L, ACMA-DA-(GG-Borax)-Ag-M and ACMA-DA-(GG-Borax)-Ag-H hydrogels with L929 cells and evaluated cell proliferation at days 1, 2, and 3. The quantitative cell survival rates showed that L929 cells in all three groups exhibited apparent proliferation varying from 1 to 3 d, with no significant differences observed between the groups (Figure 7B). Additionally, the calcein-AM/PI staining fluorescence images in Figure 7C revealed abundant green fluorescence (indicating live cells) in all groups, and most fibroblasts displayed the characteristic spindle-like morphology after incubation. These results confirmed the good proliferative activity of L929 cells, in line with the findings from the MTT assay.

Then, the hemocompatibility of the hydrogel was further investigated by testing the in vitro hemolytic activity of ACMA, ACMA-DA-(GG-Borax) and ACMA-DA-(GG-Borax)-Ag groups (Figure 7E and Figure S5, Supporting Information). The positive group of 0.1% Triton X-100 was dark red, and only the cell debris was observed under the optical microscope. Conversely, the macroscopical color of supernatants of all five biomaterial groups appeared yellowish. Meanwhile, erythrocytes in all biomaterial groups showed no significant damage and exhibited a morphology similar to that of Dulbecco’s phosphate-buffered saline (DPBS) negative control. Quantitative analysis (Figure 7D) revealed that the hemolysis rates of all hydrogels were below 5%, even for the ACMA-DA-(GG-Borax)-Ag-H sample. According to the ASTM F756-17(2025) [41], the hemolysis rates of all hydrogels are less than 5% are regarded as no hemolysis risk, which proves that biomaterials have good blood compatibility.

All these results demonstrate that the ACMA-DA-(GG-Borax)-Ag hydrogel possesses favorable hemocompatibility and cytocompatibility, which implies that they could be applied to subsequent wound treatment.

### 2.6. In Vivo Investigations of the Wound Healing Mechanism of the ACMA-DA-(GG-Borax)-Ag Hydrogel

**Figure 8:**
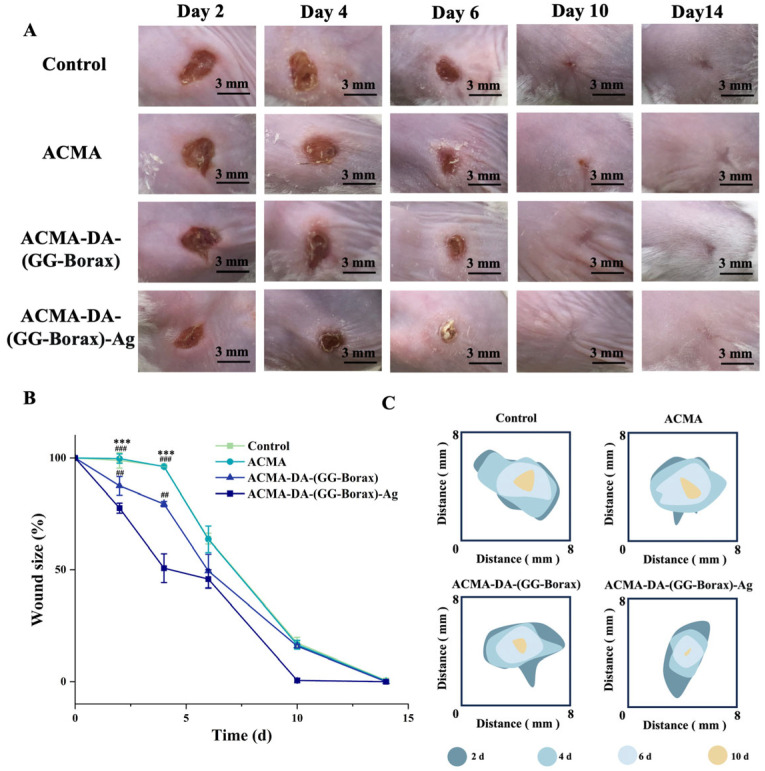
The effect of the ACMA-DA-(GG-Borax)-Ag hydrogel on the repair of full-thickness cutaneous defect in *E. coli*-infected mice. (**A**) Photographs of wound tissues on days 2, 4, 6, 10, and 14; (**B**) wound area for each group; (**C**) the diagrams of time-evolved wound areas for each group. (*n* = 6; *** *p* < 0.001; ^##^ *p* < 0.01, ^###^ *p* < 0.001.).

The results above demonstrated that the ACMA-DA-(GG-Borax)-Ag hydrogel holds great potential for promoting skin tissue repair due to its multifunctional properties. To further evaluate its antibacterial effects and wound healing capabilities in vivo, a mouse *E. coli*-infected full-thickness skin defect model was used. For comparison, phosphate-buffered saline (PBS), ACMA, ACMA-DA-(GG-Borax), and ACMA-DA-(GG-Borax)-Ag were applied to the bacteria-infected wounds. The wound areas of the mice were photographed regularly over 14 days (Figure 8A), and a quantitative analysis was performed (Figure 8B). Across all four groups, the wound areas gradually decreased over the 14-day period. The ACMA-DA-(GG-Borax)-Ag group exhibited a significantly faster wound healing rate compared to both the control and ACMA groups, as well as to the ACMA-DA-(GG-Borax) group. The wound healing speed of the ACMA-DA-(GG-Borax)-Ag group was not only significantly faster than that of the control and ACMA groups, but also superior to the ACMA-DA-(GG-Borax) group. The stacked images further confirmed the ability of ACMA-DA-(GG-Borax)-Ag to promote wound healing (Figure 8C). Specifically, the wound closure rates for the ACMA-DA-(GG-Borax) and ACMA-DA-(GG-Borax)-Ag groups were 12.50% ± 4.17% and 22.44% ± 2.27% on day 2 compared with the control (1.11% ± 1.07%) and ACMA groups (0.28% ± 0.11%). Due to their injectable properties, the ACMA-DA-(GG-Borax) and ACMA-DA-(GG-Borax)-Ag hydrogels could excellently fill the wound site, providing a conducive microenvironment for cell proliferation and epidermal growth, thereby accelerating skin regeneration. After 2 d of treatment, the wound healing in the ACMA-DA-(GG-Borax)-Ag group was progressively superior to that in the ACMA-DA-(GG-Borax) group. A significant difference in wound closure was observed between the ACMA-DA-(GG-Borax) group with or without Ag NPs on day 4. On day 6, the wounds in the ACMA-DA-(GG-Borax)-Ag groups exhibited better healing and more noticeable epidermal regeneration compared to the other three groups, highlighting the crucial role of Ag NPs in both efficiently eliminating bacteria and accelerating skin regeneration. However, the ACMA-DA-(GG-Borax)-Ag groups still had smaller open wound areas. After 10 d of treatment, the wounds in the ACMA-DA-(GG-Borax)-Ag groups achieved nearly full closure, with a wound closure rate of 98.17% ± 2.83%, showing only slight marks and more new hair growth. In contrast, the wounds in the control and ACMA groups remained open, with wound closure rates ranging from 82.78% ± 2.55% to 86.56% ± 3.01%. After 14 d of treatment, the wounds of new epidermal tissue in the ACMA-DA-(GG-Borax)-Ag groups became smooth, while the ACMA-DA-(GG-Borax) group achieved full closure, but the new epidermal tissue still showed uneven scars.

**Figure 9:**
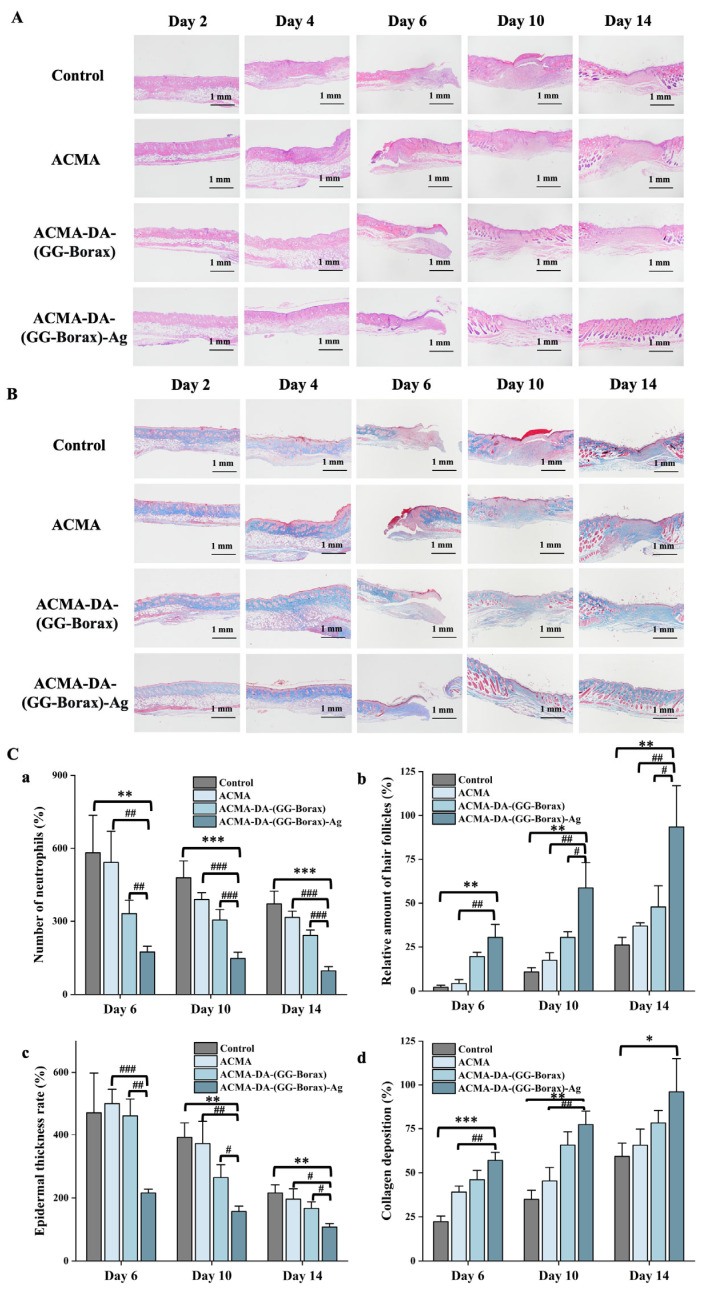
Micrographs of H&E (**A**) and Masson’s trichrome staining tissue slices (**B**) from different groups on days 2, 4, 6, 10 and 14. (**C**) Wound healing statistical data of different groups after treatment. (**a**) Number of neutrophils; (**b**) relative number of hair follicles; (**c**) epidermal thickness rate; (**d**) collagen deposition. (*n* = 6; * *p* < 0.05, ** *p* < 0.01, *** *p* < 0.001; ^#^ *p* < 0.05, ^##^ *p* < 0.01, ^###^ *p* < 0.001.)

To further assess the quality of regenerated skin in *E. coli*-infected full-thickness skin defect wounds across the four groups, tissue samples were collected and stained for histological analysis on days 2, 4, 6, 10, and 14, including hematoxylin–eosin (H&E) staining (Figure 9A) and Masson’s trichrome staining (Figure 9B) [42,43]. During the wound healing process, regenerated granulation tissues gradually filled the wound, ultimately forming dermis, epidermis, and even scab tissues. Several key indicators were used to assess healing progress [44,45,46], including the number of neutrophils (Figure 9C(a)), skin appendages like hair follicles (Figure 9C(b)), epidermal thickness rate (Figure 9C(c)) and the collagen content (Figure 9C(d)). On the 2nd and 4th days, sections of the four groups only had normal tissues for the adjacent wound. On the 6th day, the ACMA-DA-(GG-Borax)-Ag group exhibited the most pronounced granulation tissue regeneration. In all four groups, the wound tissues were covered with a new epidermal layer, although with varying degrees of defects. The control, ACMA, and ACMA-DA-(GG-Borax) groups displayed thicker epidermal layers, whereas the ACMA-DA-(GG-Borax)-Ag group showed thinner epidermal layers. Furthermore, the results of quantified neutrophils further showed that the degree of neutrophil infiltration in ACMA-DA-(GG-Borax)-Ag was significantly lower than that in the other three groups, and the introduction of Ag NPs could effectively reduce the inflammatory response. From day 6 to day 14, the number of neutrophils in the healing tissues tapered off over time, indicating a progressive reduction in the inflammatory response. In addition, compared to the control, ACMA and ACMA-DA-(GG-Borax) groups, more skin appendages could be observed in the ACMA-DA-(GG-Borax)-Ag group like hair follicles. On the 14th day, the epidermis, dermis tissues and skin appendages of the ACMA-DA-(GG-Borax)-Ag groups were almost identical to normal skin. To further assess the remodeling effect, the collagen content during the wound healing process was calculated. In the wound regions of all four groups, increased collagen deposition was observed. It was worth noting that the collagen fiber content was in the order of ACMA-DA-(GG-Borax)-Ag > ACMA-DA-(GG-Borax) > ACMA > control. Specifically, the injectable hydrogels conformed well to irregular wounds. By minimizing interfacial gaps between the wound and the dressing, they significantly enhanced sealing and moisturizing performance, providing a stable microenvironment that facilitated wound healing. Meanwhile, we confirmed that Ag NPs played a pivotal role in eradicating bacterial infection and alleviating local inflammation, thereby establishing a favorable microenvironment for tissue regeneration.

The major organs (kidneys, heart, liver, lungs, and spleen) from all four groups were harvested for biosafety assessment after 14 d of treatment (Figure S6). Neither detectable signs of damage nor inflammatory lesions were observed after 14 d. Meanwhile, the weight of these mice displayed a consistent increase throughout the entire experiment. Taken together, the above results demonstrated that the ACMA-DA-(GG-Borax)-Ag hydrogel possessed high biosafety and is a promising candidate for use as a tissue sealant to accelerate wound healing through a sutureless strategy.

## 3. Conclusions

In summary, we fabricated an injectable, self-healing, remodeling, and biocompatible multifunctional wound sealant for antibacterial and wound healing applications. We first oxidized CMA as a bio-template, and then used imine bonds to dynamically bind DA as a bio-mineralizer, resulting in the synthesis of the ACMA-DA-Ag precursor aqueous. This was followed by the addition of GG and borax to form the ACMA-DA-(GG-Borax)-Ag hydrogel in situ. The designed platforms were able to effectively achieve the reduction and stabilization of Ag^+^ and uniform loading, thereby significantly enhancing its antibacterial properties against *S. aureus* and *E. coli*. Furthermore, a multiple-dynamic-bond crosslinked network of the hydrogel improved structure stability, making it more flexible and capable of self-healing following mechanical damage, which played an active role in the wound healing process. The mouse *E. coli*–infected full-thickness skin defect model demonstrated that the hydrogel could perfectly match the irregular wound and had striking anti-infection properties and allowed for painless and quick replacement, making it particularly suitable for accelerating wound closure in a sutureless way and providing a practical integrated treatment for skin tissue reconstruction.

## 4. Materials and Methods

### 4.1. Materials

DA hydrochloride, sodium (meta) periodate (NaIO_4_) and AgNO_3_ were purchased from Sigma-Aldrich Co., Ltd., St. Louis, MA, USA. The agarose was supplied by TSINGKE, China. The ethylene glycol, isopropyl alcohol (IPA), chloroacetic acid, ethanol, sodium hydroxide and hydrogen chloride were obtained from Sinopharm Chemical Reagent Co., Ltd., Shanghai, China. The Cytotoxicity Assay Kit (CCK-8) and MTT [3-(4,5-dimethylthiazol-2-yl)-2,5-diphenyltetrazolium bromide] Cell Proliferation Kit were provided by APE x BIO Technology LLC., Houston, TX, USA. The LIVE/DEAD Cell Imaging Kit was bought from Beyotime Institute of Biotechnology, Shanghai, China. The 2.5% glutaraldehyde, Hematoxylin–Eosin/H&E Staining Kit and Masson’s Trichrome Staining Kit were purchased from Beijing Solarbio Science & Technology Co., Ltd., Beijing, China. The 4% paraformaldehyde was obtained from Wuhan Servicebio Technology Co., Ltd., Wuhan, China. Milli-Q ultrapure water (18.2 MΩ·cm; EMD Millipore, Billerica, MA, USA) was used in all experiments. All other reagents were of analytical grade and obtained commercially and used without further purification.

### 4.2. Bacterial Strains, Mice

*S. aureus* and *E. coli* were supplied by China General Microbiological Culture Collection Center (CGMCC), China.

Male BALB/c mice (7 weeks old) were purchased from the Laboratory Animal Center of the Academy of Military Medical Sciences, China.

### 4.3. Preparation of Hydrogel

Oxidization of CMA was carried out to synthesize the cross-linker ACMA via NaIO_4_ as an oxidizing agent. Briefly, NaIO_4_ aqueous solution (250 mg, 5 mL) was added dropwise into CMA ultrapure water solution (2.5%, *w*/*v*), and then the whole reaction mixture was thoroughly stirred at a speed of up to 800 rpm for 24 h in darkness at 20 °C. Afterward, ethylene glycol (2.5 mL) was added to terminate the reaction. The above mixture was dialyzed (MWCO 3500) with ultrapure water for 72 h. Finally, the purified product was freeze-dried and stored in a refrigerator until further processing.

Subsequently, ACMA was completely dissolved in Tris–HCl buffer (0.1 M, pH 7.4). DA hydrochloride (2.17 g/mL), AgNO_3_ solutions at various concentrations (0.05, 0.1, and 0.2 μg/mL), GG (0.12 g/mL), and an aqueous suspension of borax (0.06 g/mL) were then added successively. The mixture was stirred evenly until a homogeneous hydrogel was formed. These solutions were denoted ACMA-DA-(GG-Borax), ACMA-DA-(GG-Borax)-Ag-L, ACMA-DA-(GG-Borax)-Ag-M and ACMA-DA-(GG-Borax)-Ag-H.

For CMA, the agarose (5 g) was suspended in isopropyl alcohol (50 mL) at room temperature, which was stirred for 30 min. After treatment with NaOH solution (1.33 M, 50 mL), chloroacetic acid (25 g) was added to the suspension under stirring for 30 min. The resulting suspension was ultrasonicated for 30–240 min at 70 °C, then washed with 70%–100% ethanol solution, dried, and stored at 4 °C for further use.

### 4.4. Characterization

A Nicolet iS10 FTIR spectrometer (Thermo Fisher Scientific, Waltham, MA, USA) was used to characterize the FTIR spectra of the chemical structure of the samples over the range of 500–4000 cm^−1^. The background correction was performed for all spectra. A Bruker 400 MHz ^1^H-NMR spectrometer (Bruker, Billerica, MA, USA) was used to record the NMR spectra of the samples. SEM (FEI, Quanta FEG250, Hillsboro, OR, USA) images were obtained at an acceleration voltage of 20 kV. Prior to imaging, all the samples were mounted on aluminum stubs using conductive carbon tape, and sputter-coated with a platinum film to increase conductivity. Transmission electron microscope (TEM, FEI, Hillsboro, OR, USA) images of the samples were obtained using a Tecnai 20 G2 S-Twin TEM. XPS (Thermo Fisher Scientific, ESCALABA 250Xi, Waltham, MA, USA) was utilized to examine the surface chemical composition of the samples. The dried samples were evaluated in reflection mode by XRD (Bruker, D8 ADVANCE A25, Billerica, MA, USA) over a range of 2θ = 10–80°.

### 4.5. Rheological Analysis

The rheological tests of ACMA-DA-(GG-Borax)-Ag hydrogel with different solid contents were performed on a rheometer (MCR302, Anton Paar, Graz, Austria) equipped with a 25 mm-diameter parallel plate. All tests were performed at 25 °C. The frequency sweep was tested by varying the frequency (0.01–10 Hz) at 1% strain. The time scan was performed over a time range of 0 to 500 s with a fixed strain (1%) and frequency (5 Hz). The strain amplitude sweep tests were performed with an increasing shear strain from 0.005% to 1100%. The scan strain alternated between 1% and 1500%, and was held for 100 s each time. The changes in viscosity were recorded with varying shear rates from 1 to 1000 s^−1^.

### 4.6. In Vitro Antibacterial Characterizations

*S. aureus* (ATCC 6538, a Gram-positive bacterium) and *E. coli* (ATCC 8739, a Gram-negative bacterium) were used as bacterial models to evaluate the antibacterial properties of the hydrogel, and evaluated by the spread plate method.

Briefly, after sterilization the hydrogel was soaked in the diluted bacterial stock suspension with a density of about 10^5^ CFU/mL, and incubated at 37 °C for 24 h. After that, the solutions were diluted with Luria–Bertani (LB) broth using a 1000-fold dilution method, and spread evenly on a solid LB medium for incubation at 37 °C for 24 h or 48 h for *S. aureus* and *E. coli* respectively, followed by taking photographs and observing the growth of bacterial colonies. The bacteria viability without hydrogel treatment were measured and these bacteria were used as the blank group. Subsequently, the number of CFU on the agar plate was calculated. The following equation was used to further express the bacterial survival ratio:

(1) $$\mathrm{Survival}\;\mathrm{ratio}\;(\%)\;=\;\frac{\mathrm{CFU}}{{\mathrm{CFU}}_{0}}\;\times \;100\%$$

where CFU and ${\mathrm{CFU}}_{0}$ represent the number of colony-forming units with the hydrogel and without the hydrogel.

The bacteria morphologies after co-culture with hydrogel samples were examined by TEM. Briefly, bacterial suspensions were co-incubated with the hydrogel samples. The bacteria were then fixed with 2.5% paraformaldehyde at 4 °C for 24 h, dehydrated, lyophilized, and finally observed by TEM.

### 4.7. Cytocompatibility Investigation of the ACMA-DA-(GG-Borax)-Ag

The cytocompatibility investigation of the materials on the L929 cells was evaluated using the leach liquor method and MTT assay. L929 cells were seeded in 96-well plates at a density of 10,000 cells per well in complete growth medium supplemented with 10% fetal bovine serum and penicillin–streptomycin. After pre-culturing for 24 h at 37 °C in a 5% CO_2_ humidified incubator, the medium was replaced with fresh medium containing the pre-prepared material leach liquor. With the pure medium group, without the material leach liquor and serving as the control, the cells were cultured for the same time as the experimental groups. Subsequently, the cell viability was measured by typical MTT assay, and the optical density (OD) at 570 nm was measured with a microplate reader. Each sample was repeated six times (*n* = 6) and the cell viability was further expressed via the equation below:

(2) $$\mathrm{Cell}\;\mathrm{viability}\;(\%)\;=\;\frac{{\mathrm{OD}}_{e}}{{\mathrm{OD}}_{c}}\;\times \;100\%$$

where ${\mathrm{OD}}_{e}$ and ${\mathrm{OD}}_{c}$ represent the optical density of the sample group and control group, respectively.

To visualize the cell proliferation in the ACMA-DA-(GG-Borax)-Ag groups (with Ag^+^ concentrations of 0.05 to 0.1 and 0.2 μg/mL), the cells were stained via the live/dead cell imaging kit. The cells of L929 were stained with the calcein acetoxymethyl ester/propidium iodide (calcein–AM/PI) reagent mixture solution after being cultured over a period of days at temperatures varying from 1 to 3 at 37 °C in a humidified incubator, and the cell growth behavior was observed. Cell proliferation in a medium containing these materials was evaluated by the MTT assay using the same cell model.

### 4.8. Hemocompatibility Investigation of the ACMA-DA-(GG-Borax)-Ag

Hemocompatibility was evaluated by hemolysis assay. The erythrocytes were washed with DPBS three times (*n* = 3) and further diluted to a final concentration of 10% (*v*/*v*). Subsequently, the 0.1% Triton X-100 (positive control), ACMA-DA-(GG-Borax), ACMA-DA-(GG-Borax)-Ag (with the concentrations of Ag^+^ varying from 0.05 to 0.1 and 0.2 μg/mL) and DPBS (negative control) were respectively incubated with the above purified erythrocyte solution for 1 h at 37 °C. After that, all the samples were centrifuged for 10 min at 116 ×*g*, and then the supernatant was carefully transferred into a 96-well plate and measured via a microplate reader at 540 nm, respectively. All experiments were replicated 6 times (*n* = 6) for each sample. The hemolysis ratio was calculated according to the following equation:

(3) $$\mathrm{Hemolysis}\;\mathrm{ratio}\;(\%)\;=\;\frac{\left({\mathrm{OD}}_{s}-{\mathrm{OD}}_{n}\right)}{\left({\mathrm{OD}}_{p}-{\mathrm{OD}}_{n}\right)}\;\times \;100\%$$

where ${\mathrm{OD}}_{s}$, ${\mathrm{OD}}_{p}$ and ${\mathrm{OD}}_{n}$ represent the optical density of the sample and the positive and negative group, respectively.

### 4.9. In Vivo Infected Wound Healing Performance

All laboratory male BALB/c mice (7 weeks old) were randomly divided into four groups, including the control, CMA, ACMA-DA-(GG-Borax), and ACMA-DA-(GG-Borax)-Ag. After the intraperitoneal injection of 4% chloral hydrate anesthesia, the dorsal skin of all mice was depilated. Thereafter, full-thickness excisional wounds with a 6 mm diameter were constructed on the back of each mouse and clinically isolated *E. coli* was inoculated at equal quantities (1 × 10^6^ CFU/mL, 50 µL) into the wound. After the procedure, the wound sites of the mice were completely and directly covered by the prepared materials. The control group was not further treated, only secured using commercial Tegaderm (3M), and the mice were individually caged with free movement and access to food.

With the help of a phone, pictures of the wound closure were recorded at 2, 4, 6, 10 and 14 days. The dressings were renewed at the same intervals (24 h). The wound area was marked out using Image J software 1.54p, and then the stacked placement of mice wounds over time was compared and the degree of wound closure was calculated by the following formula:

(4) $$\mathrm{Wound}\;\mathrm{healing}\;\mathrm{rate}\;(\%)\;=\;\frac{\left({\mathrm{Area}}_{0}-{\mathrm{Area}}_{i}\right)}{{\mathrm{Area}}_{0}}\;\times \;100\%\;$$

where ${\mathrm{Area}}_{0}$ and ${\mathrm{Area}}_{i}$ represent the area of the initial wound and the wound on certain days, respectively.

Then, wound tissue specimens were collected from each group on days 2, 4, 6, 10, and 14 for histological evaluation. The specimens were fixed with 4% paraformaldehyde at room temperature for more than 24 h, embedded in paraffin, and sectioned for further analysis. After Masson’s trichrome staining and H&E staining of the tissue sections, the histological images were observed by an optical microscope and analyzed using Image J software to obtain the amount of collagen deposition, the epidermal thickness rate, neutrophils and the relative amount of hair follicles.

Meanwhile, the major organs, including the heart, liver, lungs, spleen and kidneys, were harvested and stained with H&E method for histological analysis to further evaluate the long-term toxicity of the hydrogel.

### 4.10. Statistical Analysis

The statistical significance was evaluated using the one-way analysis of variance with Duncan’s test for multiple comparisons. * ^(#)^, ** ^(##)^, and *** ^(###)^ represent a significant level, at 0.05, 0.01, and 0.001, respectively. All data were exhibited as mean ± standard deviation based on experiments performed in triplicate or more.

## Data Availability

The original contributions presented in this study are included in the article. Further inquiries can be directed to the corresponding authors.

## Supplementary Materials

The following supporting information can be downloaded at: https://www.mdpi.com/article/10.3390/gels12040340/s1, Figure S1: FTIR spectra of agarose and CMA; Figure S2: FTIR spectra (A) and ^1^H-NMR spectra of (B) CMA and ACMA; Figure S3: Photograph of the ACMA-DA mixture; Figure S4: Degrees of swelling (A) and degradation (B) of hydrogel; Figure S5: Photograph of the hemolysis assay (a. DPBS; b. ACMA; c. ACMA-DA-(GG-Borax); d. ACMA-DA-(GG-Borax)-Ag-L; e. ACMA-DA-(GG-Borax)-Ag-M; f. ACMA-DA-(GG-Borax)-Ag-H; g. Triton X-100); Figure S6: Micrographs of H&E-stained major organ tissue slices from mice from different groups after 14 d of treatment; Movie S1: Adhesive behaviors of the ACMA-DA-(GG-Borax)-Ag hydrogel; Movie S2: Injectable properties of the ACMA-DA-(GG-Borax)-Ag hydrogel; Movie S3: Removability of the ACMA-DA-(GG-Borax)-Ag hydrogel.

## Abbreviations

The following abbreviations are used in this manuscript:

^1^H-NMR	^1^H-Nuclear Magnetic Resonance
ACMA	Aldehyde Carboxymethylated Agarose
CFU	Colony-Forming Unit
CMA	Carboxymethylated Agarose
DA	Dopamine
DPBS	Dulbecco’s Phosphate-Buffered Saline
*E. coli*	*Escherichia coli*
FTIR	Fourier Transform Infrared Spectroscopy
GG	Guar Gum
H&E	Hematoxylin–Eosin
OD	Optical Density
PBS	Phosphate-Buffered Saline
*S. aureus*	*Staphylococcus aureus*
SEM	Scanning Electron Microscope
TEM	Transmission Electron Microscope
XPS	X-ray Photoelectron Spectroscopy
XRD	X-ray Diffraction
