# Supplementary material for: Multiple-Dynamic-Bond Cross-Linked Injectable Antibacterial Hydrogel Sealants with Self-Healing for Wound Healing
Source: Gels. 2026 Apr 19;12(4):340. doi: 10.3390/gels12040340 (PMC13115437; doi:10.3390/gels12040340)
Supplement: Supplementary file 1 [file gels-12-00340-s001.zip › gels-4237408-supplementary/Supplementary Files/Supplementary File.pdf]

[Supplementary Information]

Article

Multiple-Dynamic-Bond Cross-Linked Injectable Antibacterial Hydrogel Sealants with Self-Healing for Wound Healing

Tingting Wei [1], Yunrui Cao [2], Shuo Yang [1], Yu Song [1], Yanjun Liu [1], Hu Hou [1,3], Jie Xu [1,3,*] and Changhu Xue [1,3,4,*]

[1] State Key Laboratory of Marine Food Processing & Safety Control, College of Food Science and Engineering, Ocean University of China, No. 1299, Sansha Road, Qingdao 266404, China; ttwei621@163.com (T.W.); yangshuo19988183@163.com (S.Y.); songyu@ouc.edu.cn (Y.S.); liuyanjun@ouc.edu.cn (Y.L.); houhu@ouc.edu.cn (H.H.)

[2] Optoelectronic Information Center, Taizhou Institute of Zhejiang University, Taizhou 318000, China; caoyunrui2024@zju.edu.cn (Y.C.)

[3] Sanya Ocean Institute, Ocean University of China, Sanya 572000, China

[4] Qingdao Marine Science and Technology Center, Qingdao 266235, China

* Correspondence: xujie9@ouc.edu.cn (J.X.); xuech@ouc.edu.cn (C.X.)

As shown in Figure S1, in the FTIR spectrum of agarose, a strong absorption peak at 3428 cm⁻¹ is attributed to the O-H stretching vibration. The peak at 2898 cm⁻¹ corresponds to the C-H stretching vibration, and the characteristic absorption peak at 1077 cm⁻¹ is assigned to the C-O-C stretching vibration [22]. Furthermore, in comparison to unmodified agarose, the FTIR spectrum of CMA exhibited minimal differences, with the sole exception being the emergence of a distinct absorption peak corresponding to the carbonyl (C=O) stretch of the carboxyl group (-COOH) at 1744 cm⁻¹ [22]. Collectively, these findings led to the preliminary confirmation of the successful preparation of CMA. The degree of substitution of the prepared CMA was determined via acid-base titration, giving a DS value of 74.1%.

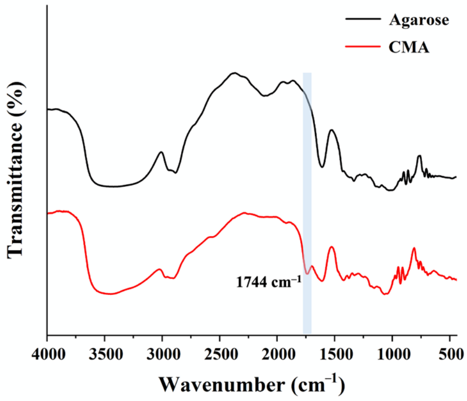

Figure S1. FTIR spectra of agarose and CMA.

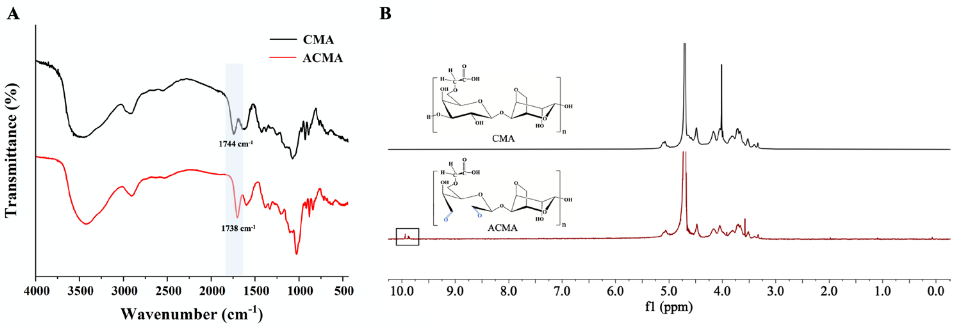

Figure S2. FTIR spectra (**A**) and ^{1}H-NMR spectra (**B**) of CMA and ACMA.

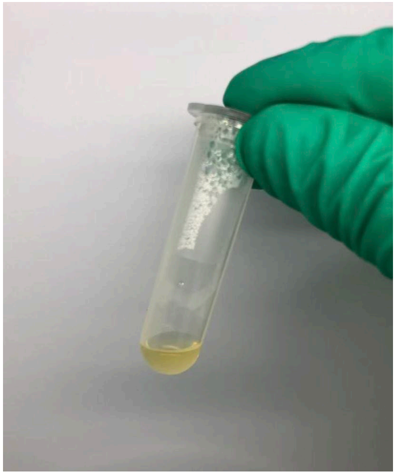

Figure S3. Photograph of the ACMA-DA mixture.

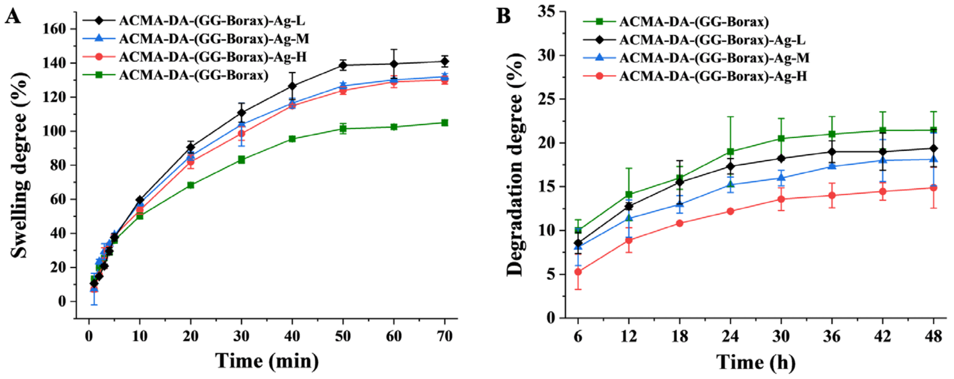

Figure S4. Degrees of swelling (**A**) and degradation (**B**) of hydrogel.

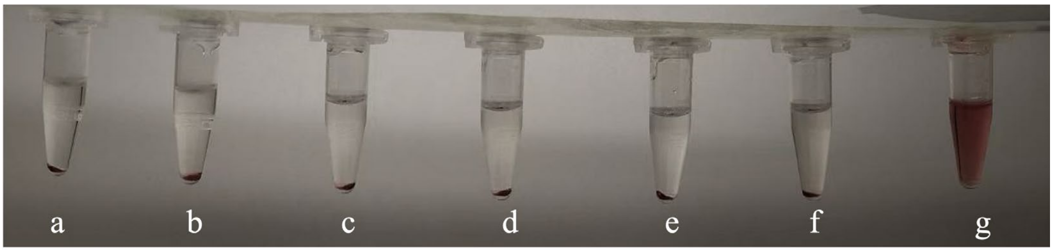

Figure S5. Photograph of the hemolysis assay (**a**. DPBS; **b**. ACMA; **c**. ACMA-DA-(GG-Borax); **d**. ACMA-DA-(GG-Borax)-Ag-L; **e**. ACMA-DA-(GG-Borax)-Ag-M; **f**. ACMA-DA-(GG-Borax)-Ag-H; **g**. Triton X-100).

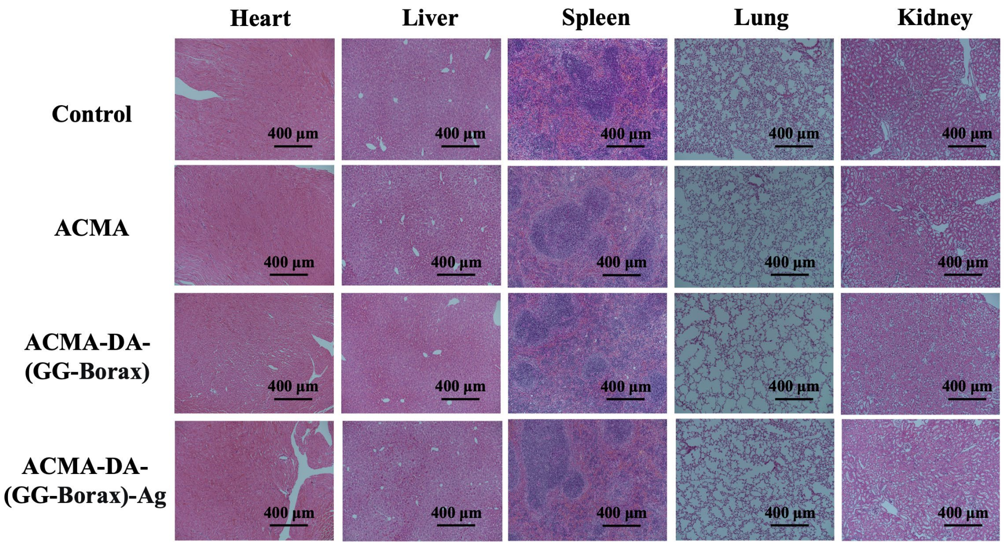

Figure S6. Micrographs of H&E-stained major organ tissue slices from mice from different groups after 14 d of treatment.
